# Digital Survey–Based Tracing of COVID-19 Over the Early Pandemic: Comprehensive Geospatial and Symptomatic Analysis in Lebanon

**DOI:** 10.2196/80331

**Published:** 2025-11-20

**Authors:** Youssef Bassim, Abir Abdelrahman, Amal Iaaly, Gavin M Douglas, Patrick Daou, Mayda Finianos, Rim Hassan, Ibrahim Nassif, Layal Greige, Liza Dib, Jean Marc Mardirossian, Mira El Chaar

**Affiliations:** 1 Faculty of Medicine, University of Balamand, Koura, Lebanon; 2 Faculty of Health Sciences, University of Balamand, Rond Point Saloumeh-Dekouaneh, P.O. Box: 55251 Sin El Fil, Beirut, 55251, Lebanon, 961 1 495833; 3 Faculty of Engineering, University of Balamand, Koura, Lebanon; 4 Department of Biology, University of New Brunswick, Fredericton, NB, Canada; 5 Department of Nephrology and Hypertension, Mayo Clinic, Jacksonville, FL, United States

**Keywords:** COVID-19, community engagement, digital health, GIS, Lebanon, risk stratification, surveillance, geographic information system

## Abstract

**Background:**

In response to the early spread of COVID-19 in Lebanon, the University of Balamand developed the HAYATI app, a community-focused, geographic information system (GIS)–based digital health platform aimed at enhancing public health surveillance. At the time, while the Lebanese Ministry of Public Health utilized centralized dashboards to report confirmed cases and monitor national trends, no interactive tool existed to engage the public directly in real-time risk assessment and surveillance, especially in underserved regions.

**Objective:**

The aim of this study was to design, implement, and evaluate the effectiveness of the HAYATI app as a GIS-integrated digital surveillance tool to identify high-risk individuals and support targeted testing and contact tracing during the early stages of the COVID-19 pandemic in Lebanon.

**Methods:**

The HAYATI app was launched in March 2020 using ArcGIS Survey123 and real-time dashboards, incorporating a risk scoring algorithm based on 21 clinical and behavioral criteria. Between April 2020 and March 2021, self-reported data were collected from 10,235 individuals across Lebanon. Participants identified as high or major risk through the automated scoring algorithm were referred for free polymerase chain reaction testing at the University of Balamand. Test results were securely communicated to local municipalities and the Ministry of Public Health. Data were analyzed for associations between symptoms and positivity rates, as well as geographic and demographic trends using spatial analysis tools.

**Results:**

Of the 10,235 individuals who submitted data, 1782 were classified as high or major risk and referred for polymerase chain reaction testing. Among them, 394 (22.1%) tested positive for SARS-CoV-2. Loss of smell and taste was strongly associated with positive test results (*P*<.001). The highest positivity rates were observed among individuals aged 18‐29 years and in the North Governorate. GIS mapping enabled real-time visualization of case clusters, which informed localized containment responses.

**Conclusions:**

The HAYATI app effectively filled a critical surveillance gap during the early pandemic phase in Lebanon. By integrating GIS technology, automated risk stratification, and community-level engagement, it provided a scalable model for public health surveillance in resource-limited settings. This approach has potential for broader applications in managing future outbreaks and endemic diseases through decentralized, real-time digital health strategies.

## Introduction

**Figure 1.**
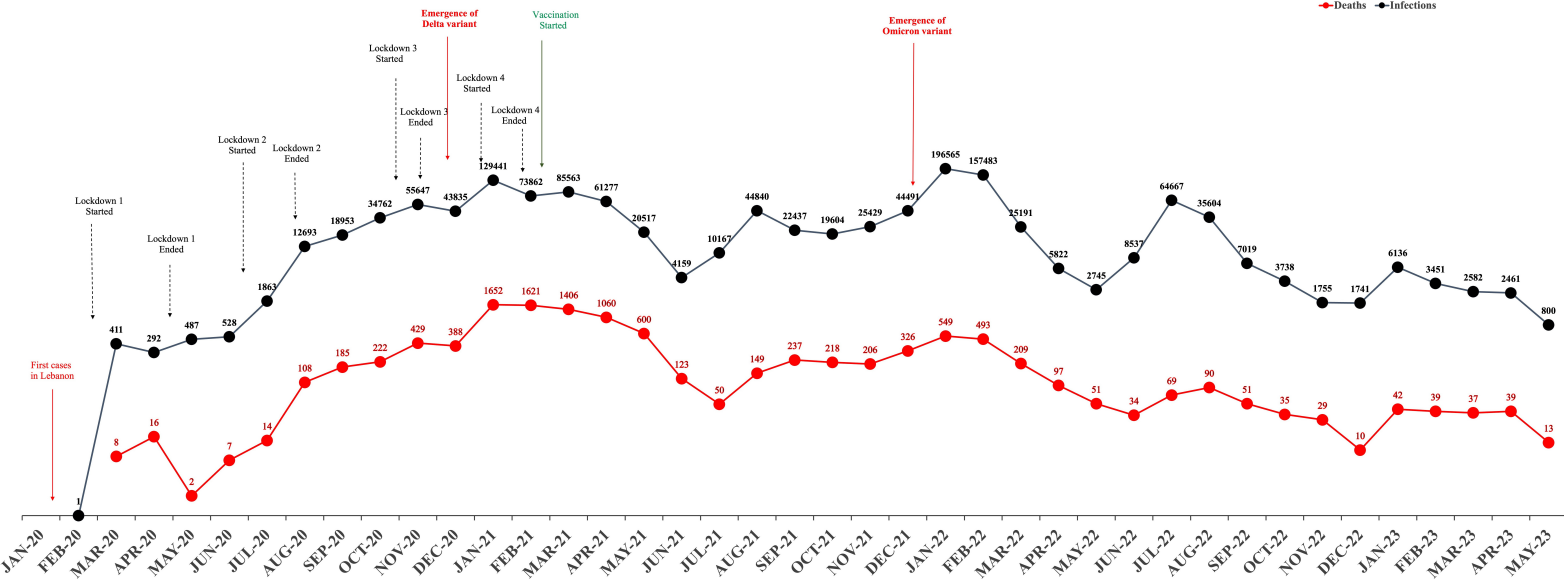
Monthly trends of infection and death cases with key pandemic events and interventions (January 2020-May 2023). The black line indicates active cases, while the red line denotes deaths, with each point on the lines corresponding to the number of cases reported monthly. Key events are marked, including the start and end of 4 lockdown periods, the emergence of the Delta and Omicron variants, and the beginning of vaccination efforts. Following May 2023, the data for both active cases and deaths are considered incomplete due to the discontinuation of regular reporting by centers, which no longer publish positivity rate statistics.

The emergence of the novel coronavirus SARS-CoV-2 in Wuhan, China, in December 2019, set off a chain of events that would lead to a global health emergency unprecedented in recent history [1-3]. The first confirmed case of COVID-19 in Lebanon, reported on February 21, 2020, marked the beginning of the nation’s encounter with the pandemic, leading to a series of public health responses aimed at curbing the virus’s spread [4]. Lebanon’s COVID-19 epidemiological trajectory was characterized by fluctuating infection and mortality rates, a pattern mirrored by public health responses that ranged from stringent lockdowns to comprehensive vaccination campaigns (Figure 1). The challenges faced by the government of Lebanon in response to the emerging pandemic were enormous due to very limited resources as the outbreak came at a time when Lebanon was going through the worst economic crisis in its history and during an unstable and conflicting political situation. The coronavirus outbreak also stretched the health sector that was already strained due to the lack of finance caused by the banking crisis that imposed severe restrictions on foreign currency fund transfers from the Lebanese Central Bank. This has caused a setback in Lebanon’s effort in its fight against COVID-19 on top of all other economic disasters. Despite all the efforts taken by the Lebanese government to mobilize resources to equip public hospitals, the unmet needs were immense and the hospitals remained underequipped. At the start of the pandemic, COVID-19 testing was limited to a single designated facility at Rafic Hariri Public Hospital in Beirut, with a restricted supply of testing kits that could not meet the high market demand. Parallel to that, there were concerns that the coronavirus outbreak would hit vulnerable areas (poor and overpopulated) in Lebanon, such as the North Governorate, where people could not afford polymerase chain reaction (PCR) testing, and government intervention was limited in combating the spread of the pandemic. Given the limited testing capacity and resources, it became necessary to develop a targeted testing strategy to rationalize and prioritize the use of PCR kits. This included the smart identification of high-risk individuals, the establishment of a contact tracing mechanism, and the implementation of a robust surveillance system. In response to these challenges, the GIS Center at the Faculty of Engineering at the University of Balamand harnessed the power of geographic information systems (GISs) to enable rapid screening and tracing of COVID-19 cases, while also supporting the targeted use of PCR testing. By mid-March 2020, just 2 weeks after the first confirmed case in Lebanon, the GIS Center launched the HAYATI app, built using the Esri Geospatial Cloud. During this time, the strategic application of GIS emerged as an essential tool in navigating the complexities introduced by the pandemic [5]. GIS played a pivotal role in tracking and tracing the spread of COVID-19 by enabling spatial visualization, real-time data integration, and informed decision-making. Through interactive dashboards, GIS facilitated the monitoring of infection rates, mortality, and recovery data across global, national, and local scales. Health authorities used GIS tools to identify hotspots, track the movement of infected individuals, and model the spread of the virus, which was critical in guiding quarantine efforts and allocating medical resources. Additionally, GIS supported contact tracing initiatives by integrating spatial and temporal data, helping to map the networks of potential exposure. By linking demographic, mobility, and health data, GIS allowed for targeted interventions and effective communication with the public, underscoring its value as an essential tool in public health crisis management.

In this study, we highlight how the HAYATI app provided valuable insights into the interplay between clinical symptoms, demographics, and public health interventions, demonstrating the effectiveness of GIS in characterizing the pandemic. We also discuss the continued relevance of digital health tools like the HAYATI project in managing the endemic phase of COVID-19 and emphasize their critical role in addressing current and future public health challenges.

## Methods

### The HAYATI App

By mid-March 2020, just 2 weeks after the first COVID-19 case was reported in Lebanon, the GIS Center at the Faculty of Engineering at the University of Balamand launched the HAYATI app, a digital public health solution built using the Esri Geospatial Cloud. The app was developed as part of the University’s social responsibility efforts to support national containment strategies, particularly in underserved areas of North Lebanon. It was made freely accessible to municipalities across the country to support the rapid identification and management of COVID-19 cases.

**Figure 2.**
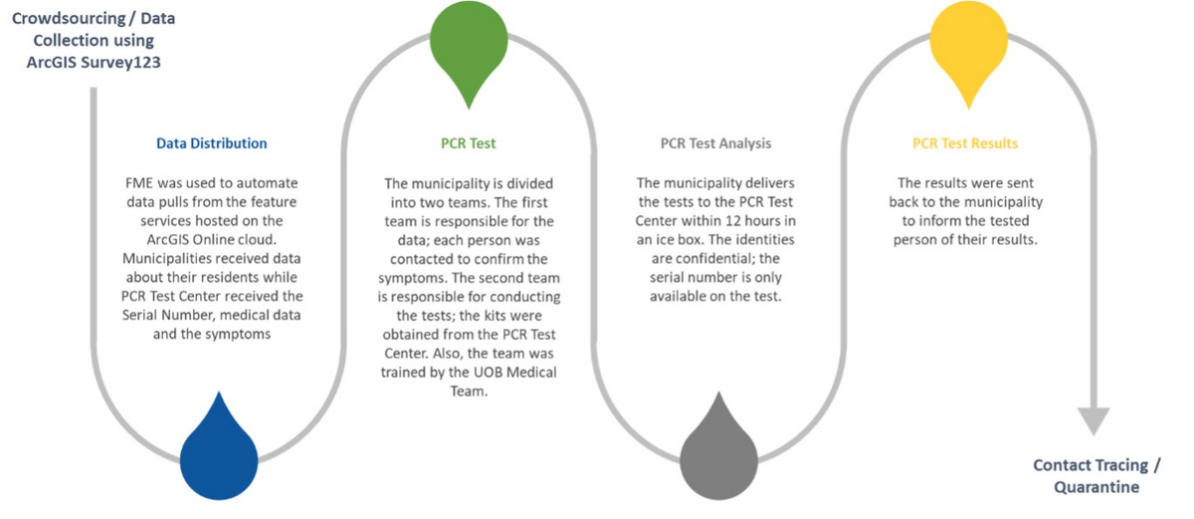
Operational workflow of the HAYATI app for COVID-19 risk assessment and targeted testing. This figure outlines the end-to-end workflow implemented through the HAYATI app, developed by the University of Balamand to support targeted polymerase chain reaction (PCR) testing and contact tracing during the COVID-19 pandemic. The process begins with data crowdsourcing using ArcGIS Survey123, where respondents provide information related to symptoms, demographics, and risk factors. Feature Manipulation Engine (FME) automates the extraction of these data from ArcGIS Online and distributes the data to municipalities and the PCR testing center. Municipalities divide their teams: one validates symptom data and contacts individuals, while the second handles logistics for PCR sample collection using kits provided by the University of Balamand (UOB) medical team. Samples are transported to the PCR testing center within 12 hours for analysis, with patient identities protected by serial number coding. Test results are reported back to municipalities, which then notify individuals and initiate contact tracing or quarantine measures based on the outcomes.

The HAYATI app integrated core Esri technologies, including ArcGIS Survey123 (version 3.9.149) for smart data collection and ArcGIS Dashboards for real-time data visualization and sharing. To streamline data processing, the Feature Manipulation Engine (version 2020.2.0.0; Safe Software Inc.) was used to extract and automate data transfers from ArcGIS Online feature services. The Esri ArcGIS Portal Feature Service was further embedded into the Feature Manipulation Engine pipeline to ensure timely dissemination of collected data to both municipal stakeholders and the University of Balamand’s PCR Testing Center (Figure 2). This fully coordinated system created a closed-loop workflow involving municipalities, the PCR test center, and the University of Balamand medical team. Figure 2 illustrates the end-to-end process, from data crowdsourcing and distribution to PCR testing, laboratory analysis, result reporting, and follow-up actions such as quarantine and contact tracing. This workflow enabled efficient identification, risk-based triage, and timely intervention for individuals likely to have contracted COVID-19.

At the core of the app was a COVID-19 risk calculator developed by the University of Balamand, in line with recommendations from the World Health Organization, the Centers for Disease Control and Prevention, and the Lebanese Society of Infectious Diseases and Clinical Microbiology. The calculator incorporated 21 criteria grouped into 3 categories. Group 1 (critical exposures and symptoms) included 5 criteria: recent travel, contact with a traveler, contact with a suspected case, and sudden loss of smell or taste. The presence of any single Group 1 criterion automatically classified the respondent as high risk and mandated referral for PCR testing. Group 2 (key clinical symptoms) included 4 criteria: dry cough, sore throat, fever, and shortness of breath. The presence of any 2 Group 2 criteria also triggered PCR referral. Group 3 (additional epidemiological and clinical risk factors) included 12 criteria: public transportation use, exposure to crowded areas, hospital visits, rhinorrhea, productive cough, common cold, diarrhea, myalgia, headache, fatigue, abdominal pain, and cyanosis. Each of these criteria was assigned a weight ranging from 1 to 3 points. When neither Group 1 nor Group 2 conditions were met, the cumulative weight of Group 3 criteria determined the overall risk category.

Based on the total score, individuals were stratified into 4 categories: minor (<10% of total weight; score 0‐2), moderate (10%‐30%; score 2‐6), major (30%‐60%; score 6‐12), and high (>60%; score 12‐20) (Table 1). Only those categorized *as* major *or* high *risk* were referred for free PCR testing at the University’s testing center. This targeted testing strategy was crucial during the country’s economic crisis, as it enabled rational allocation of limited testing kits and resources. The full list of variables and their assigned weights is provided in Table S1 in Multimedia Appendix 1, while Table 1 summarizes the scoring thresholds and corresponding testing recommendations.

**Table 1.** COVID-19 risk score calculation and polymerase chain reaction (PCR) testing strategy.

Risk level	Minimum risk score	Maximum risk score	PCR test
Minor	0	2	No PCR test
Moderate	2	6	No PCR test
Major	6	12	PCR recommended
High	12	20	PCR needed

**Figure 3.**
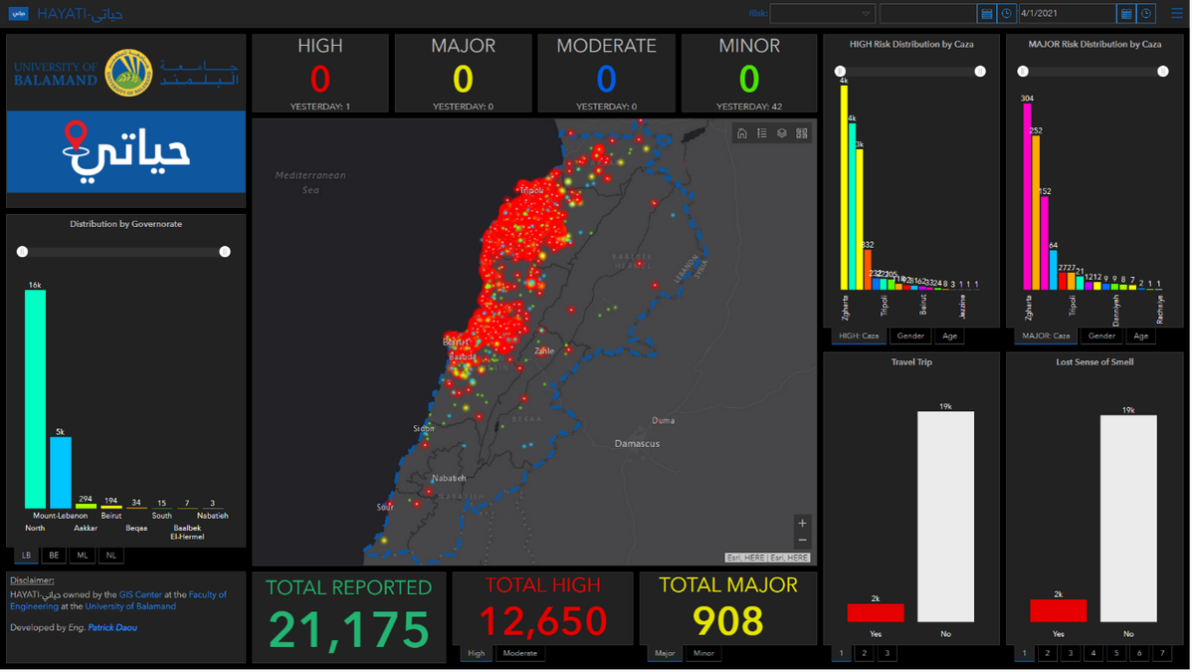
Visualization of COVID-19 risk distribution via the HAYATI dashboard. This figure presents a comprehensive screenshot of the HAYATI dashboard developed by the GIS Center at the University of Balamand, showcasing the geographic and demographic distribution of COVID-19 risk across Lebanon. The central map displays the locations of respondents, with color-coded dots representing different risk levels: red for high risk, orange for major risk, yellow for moderate risk, and green for minor risk. At the top of the dashboard, the current number of high, major, moderate, and minor risk cases is displayed, with a comparison to the previous day’s data. On the left sidebar, the distribution of cases by governorate highlights the regions most affected. To the right of the map, bar charts illustrate the breakdown of high and major risk cases by gender, age group, and caza (district). Additional charts below include data on respondents’ travel history and the presence of loss of smell, a key symptom of COVID-19. At the bottom of the dashboard, summary statistics show the total number of reported respondents (n=21,175), high-risk cases (n=12,650), and major-risk cases (n=908).

To complement the risk stratification system, an operational dashboard was developed using ArcGIS to display risk scores and testing outcomes in real time (Figure 3, Figure S1 in Multimedia Appendix 2). The dashboard provided geographic visualization of COVID-19 risk levels by plotting respondent data on an interactive map. Users could monitor daily changes in case distribution and view demographic breakdowns (by age, gender, and district) of high-and major-risk respondents. It also displayed epidemiological indicators such as recent travel history and hallmark COVID-19 symptoms (eg, fever, cough, loss of smell), alongside aggregated PCR test results at the municipal level.

Beyond its real-time monitoring capabilities, the HAYATI app was uniquely designed to integrate risk prediction, testing triage, and surveillance within a single platform. Unlike Go.Data, a digital tool developed by the WHO that focuses primarily on case investigation and contact follow-up, the HAYATI app combined algorithm-based risk scoring with geospatial visualization and diagnostic referral. This integration enabled immediate classification and response, ensuring that high-risk individuals received timely diagnostic testing and public health follow-up.

### Participant Enrollment and App Accessibility

The app was primarily developed to support surveillance efforts in North Lebanon; however, it was made publicly accessible to all individuals across Lebanon, without geographic restriction. Any person residing in Lebanon could access the app, complete the survey, and be referred for testing, regardless of their governorate of residence. Our survey app was deployed between April 2020 and March 2021, during the first and second waves of the COVID-19 pandemic in Lebanon.

### Nucleic Acid Extraction and SARS-CoV-2 Testing

Sample collection began with a nasopharyngeal swab from each participant. The swab was immediately placed into viral transport medium and transported to the Microbiology and Molecular Biology Laboratory at the University of Balamand, a facility certified for COVID-19 testing and operating in coordination with the Lebanese Ministry of Public Health. Upon arrival, SARS-CoV-2 RNA extraction was performed using the AccuPrep Viral RNA Extraction Kit. Extracted RNA was then subjected to real-time reverse transcription polymerase chain reaction testing using the GeneFinder COVID-19 Plus RealAmp Kit, which targets the nucleocapsid, envelope, and RNA-dependent RNA polymerase genes of the virus. RNase P was used as an internal control to ensure sample integrity and extraction efficiency.

### Data Processing

Although no age restrictions were applied during participant recruitment, we performed several quality-control filters on the survey data to ensure data consistency and reliability for analysis. First, we removed 13 entries that shared the same participant serial number, retaining only 1 representative response per duplicate set. We then excluded 60 participants under 5 years old and 3 participants over 90 years old. These extreme age values were removed due to concerns about data accuracy and the ability of very young participants to self-report symptoms reliably. After filtering, a total of 10,954 participant entries were retained for downstream analyses.

### Statistical Analysis

Statistical analyses were performed using the R programming language (v4.2.2; R Core Team) [6]. Enriched demographic factors and symptoms in SARS-CoV-2 positive versus negative individuals were identified based on Fisher exact tests, implemented via the exact2x2 R package (v1.6.9) [7]. In cases where frequency tables larger than 2×2 were analyzed, Fisher exact tests were performed using Monte Carlo simulations with the fisher.test function from the stats package. All symptoms were initially tested individually, and statistical significance was determined using Benjamini-Hochberg-adjusted *P* values, with a threshold of <.05. For the multisymptom analysis, we focused on symptom combinations that were observed in at least 10 participants to ensure sufficient statistical power. This more lenient threshold was adopted due to decreased statistical power and was considered justified as the analysis aimed to recapitulate well-known symptomatic signatures based on digital survey data rather than to robustly identify novel associations.

We conducted a PERMANOVA (permutational multivariate analysis of variance) using the adonis2 function from the vegan R package (v2.6.4) [8], based on pairwise Jaccard distances computed from the symptom presence or absence matrix. Only individuals reporting at least 2 symptoms were included in this analysis.

All data visualizations were also generated in R. Heatmaps were produced using the ComplexHeatmap package (v2.14.0) [9], and UpSet plots were generated using ComplexUpset (v1.3.3) [10]. Shapefiles representing Lebanese administrative boundaries were downloaded from the Humanitarian Data Exchange [11], maintained by the Office for the Coordination of Humanitarian Affairs. All additional figures were created using the ggplot2 package (v3.4.0) [9]. The complete analysis code is publicly available on GitHub [12].

### Ethical Considerations

A pivotal aspect of the Hayati project’s integrity and commitment to ethical standards was its approval by the institutional review board at the University of Balamand (IRB-REC/o/023-07/1123). This approval highlights the project’s strict adherence to ethical research principles, ensuring that all data collection and participant engagement were conducted responsibly and with full respect for individual privacy and welfare.

Participation in the Hayati study was open to the public and entirely voluntary. Individuals who suspected exposure to COVID-19, experienced COVID-19-like symptoms, or were simply concerned about their health status were encouraged to participate. There were no specific inclusion or exclusion criteria, and no age restrictions were imposed. Any person willing to undergo screening and complete the survey was eligible. In cases where the participant was a minor, the application was filled out by a parent or legal guardian, and children were accompanied by a guardian during testing and enrollment. Recruitment occurred through community outreach in coordination with municipalities, health care centers, and public awareness campaigns.

All participants provided informed consent through the application, which clearly described the purpose of the study, procedures involved, potential risks and benefits, and strict confidentiality measures. Data collected via the app were securely stored in the ArcGIS online cloud platform, with full compliance with Lebanese data protection laws. Data access and processing were handled solely by the University of Balamand, with offline backup and secure archiving maintained at the GIS Center within the Faculty of Engineering.

## Results

### Demographic Distribution by Region, Gender, and Age

**Figure 4.**
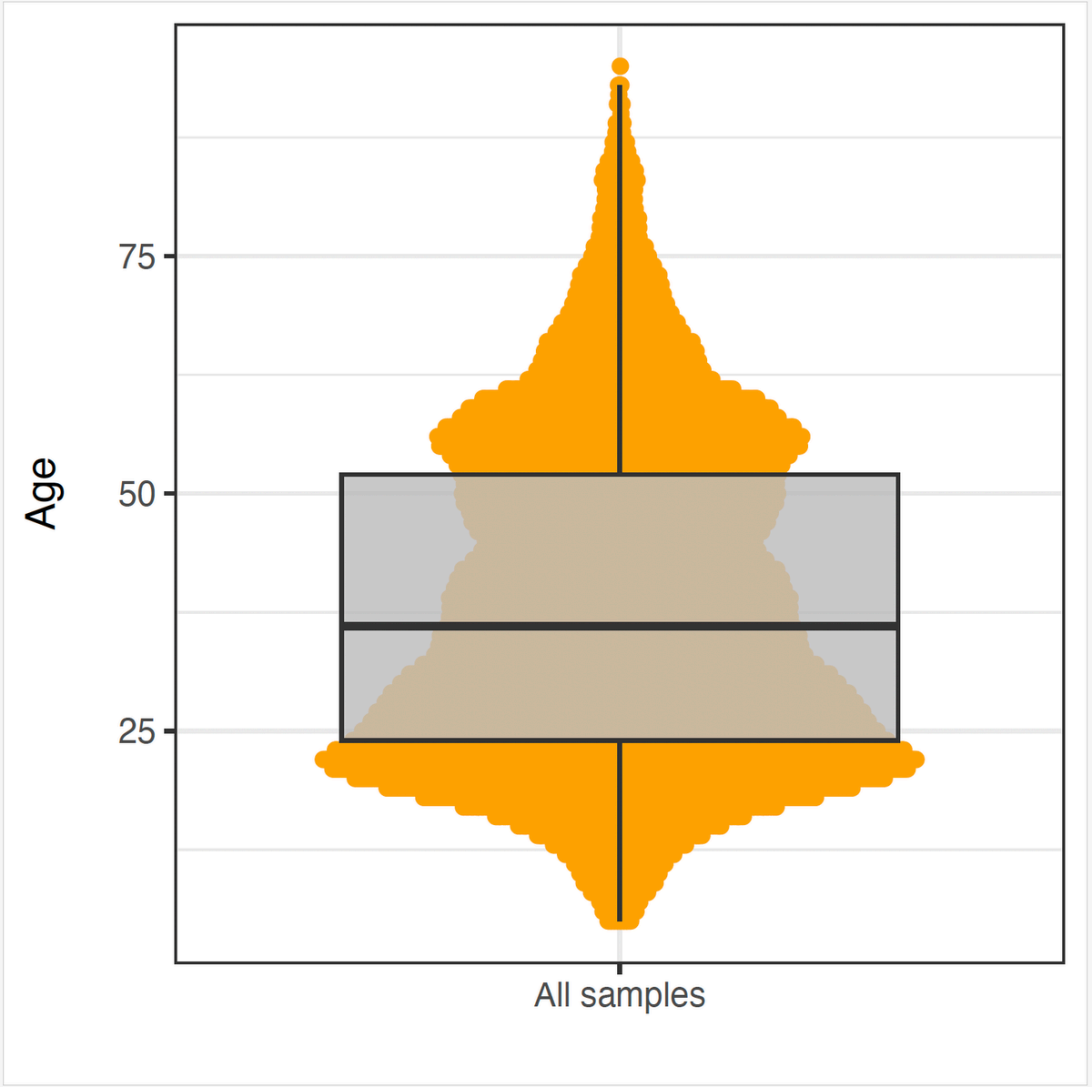
Age distribution of all samples tested for COVID-19. The violin plot illustrates the density of different ages, showing both the range and the frequency of ages within the tested population. The boxplot within the violin plot provides a summary of the central tendency and dispersion of ages, with the median age indicated by the thick black line.

Our filtered dataset includes results from 10,954 individuals tested for COVID-19. Table 2 summarizes the demographic statistics of these individuals, categorized by governorate, gender, and age group. The largest proportion of individuals resided in the North governorate, ~70% (n=7635) of the population, followed by Mount Lebanon with ~25% (n=2781). The gender distribution was balanced, with males (n=5640, 51.5%) slightly outnumbering females (n=5116, 46.7%). Age information was available for the majority of participants; however, 42 individuals did not provide their age and were therefore excluded from age-specific analyses but retained in other parts of the dataset. Among those with available age data, the largest group was aged 18-29 years, indicating a youthful demographic, while those aged above 90 constituted the smallest group. The mean age of participants with known age was 38 years (SD=17) (Figure 4).

**Table 2.** Demographic and clinical characteristics of polymerase chain reaction (PCR)–tested individuals across governorates in Lebanon.

	Total, n (%)	Positive for SARS-CoV-2, n (%)
Governate		
Akkar	411 (3.8)	37 (9)
Baalbek-Hermel	2 (0.0)	0 (0)
Beirut	103 (0.9)	6 (5.8)
Beqaa	15 (0.1)	1 (6.7)
Mount Lebanon	2781 (25.4)	319 (11.5)
El Nabatieh	1 (0.0)	0 (0)
North	7635 (69.7)	907 (11.9)
South	6 (0.1)	0 (0)
Missing	0 (0)	0 (0)
Gender		
Female	5116 (46.7)	605 (11.8)
Male	5640 (51.5)	639 (11.3)
Missing	198 (1.8)	26 (13.1)
Blood group		
A+	3278 (29.9)	465 (14.2)
B+	784 (7.2)	96 (12.2)
AB+	299 (2.7)	40 (13.4)
O+	2908 (26.5)	360 (12.4)
A–	263 (2.4)	40 (15.2)
B–	72 (0.7)	11 (15.3)
AB–	44 (0.4)	10 (22.7)
O–	325 (3)	37 (11.4)
Missing	2981 (27.2)	211 (7)
Social distancing		
Yes	4370 (39.9)	427 (9.8)
No	6525 (59.6)	840 (12.9)
Missing	59 (0.5)	3 (5.1)
Public transportation		
Yes	1530 (14)	124 (8.1)
No	9420 (86)	1146 (12.2)
Missing	4 (0.0)	0 (0)
Crowded area		
Yes	3599 (32.9)	317 (8.8)
No	7351 (67.1)	953 (13.0)
Missing	4 (0.0)	0 (0)
Recent travel		
Yes	1141 (10.4)	25 (2.2)
No	9809 (89.5)	1245 (12.7)
Missing	4 (0.0)	0 (0)
Contact with travelers		
Yes	653 (6)	28 (4.3)
No	10,297 (94)	1242 (12.1)
Missing	4 (0.0)	0 (0)
Contact with COVID positive patients		
Yes	4133 (37.7)	595 (14.4)
No	6819 (62.3)	675 (9.9)
Missing	2 (0.0)	0 (0)
Hospital visit		
Yes	941 (8.6)	84 (8.9)
No	10,009 (91.4)	1186 (11.8)
Missing	4 (0.0)	0 (0)

### Associations Between Demographic and Behavioral Factors With SARS-CoV-2 Positivity Rates

Our dataset provides a detailed breakdown of SARS-CoV-2 positivity rates across various demographic and behavioral factors, including gender, age group, geographic region, quarantine practices, and blood group, as illustrated in Table 2. Overall, the COVID-19 positivity rate was approximately 11.5% (1270/10,954). Rates were comparable between females (605/5116, 11.8%) and males (639/5640, 11.3%), indicating no significant difference by gender.

While age groups showed relatively consistent positivity rates, slightly higher rates were observed among participants aged 30‐39 years. In contrast, more pronounced differences appeared across blood group types, with the lowest positivity rate among individuals with blood type O– (37/325, 11.4%) and the highest among those with AB– (10/44, 22.7%). These trends prompted further statistical analysis of the association between blood group and SARS-CoV-2 positivity, presented below.

To assess the relationship between blood group and COVID-19 test positivity, we performed Fisher exact tests, comparing the distribution of positive and negative cases for each blood group against all other groups combined. A statistically significant association was identified: individuals with blood type O+ were significantly less likely to test positive (OR 0.716, 95% CI 0.72-0.93; BH-corrected *P*=.01).

We also tested for associations between SARS-CoV-2 positivity and 7 self-reported behavioral factors, all of which showed statistically significant associations (BH-corrected *P*<.006, Fisher exact tests). Two behaviors followed expected patterns: individuals who reported adhering to social distancing had a lower positivity rate (427/4370, 9.8%) compared to those who did not (840/6525, 12.9%), and those who reported contact with confirmed COVID-19 cases had a higher positivity rate (595/4133, 14.4%) compared to those without known contact (675/6819, 10.9%).

In contrast, additional behaviors, which would be expected to be associated with higher positivity rates (were all possible confounders excluded), were significantly associated with lower positivity rates. In particular, the use of public transportation was associated with an 8.1% (124/1530) positivity rate, whereas those not using public transportation had a higher rate of 12.2% (1146/9420). Being in crowded areas was associated with a lower positivity rate of 8.8% (317/3599) compared to a 13.0% (953/7351) positivity rate for those who avoided crowded areas. Similarly, recent travel was negatively related to positivity rates, with recent travelers showing a rate of 2.2% (25/1141) against 12.7% (1245/9809) for those who had not traveled. In addition, individuals who had contact with travelers showed a lower COVID-19 positivity rate (28/653, 4.3%) compared to those who did not have such contact (1242/10,297, 12.1%). Separately, participants who reported visiting a hospital recently had a positivity rate of 8.9% (84/941), whereas those who had not visited a hospital had a higher rate of 11.8% (1186/10,009).

### Age-Specific Analysis of COVID-19 Positivity Rates

In the analysis of COVID-19 positivity rates across different age groups, we observed notable variation in positivity percentages (Multimedia Appendix 3). The study included participants aged 5 years and above, following quality-control filtering that excluded individuals under the age of 5 years. Age groups ranged from 5 to over 90 years, with data analyzed accordingly.

A total of 1270 PCR-positive cases were identified from the tested individuals. The age-specific positivity rates highlight variation in infection distribution across different age groups (Multimedia Appendix 3). Notably, higher positivity rates were observed among individuals aged 11‐17 years and those over 80 years old, compared to other age cohorts. For example, the 11‐17 age group exhibited a higher positivity rate than younger children. In contrast, younger adults and middle-aged groups showed relatively lower positivity rates. These patterns were recorded during the study period, which coincided with phases of school closures, lockdowns, and movement restrictions in Lebanon. While individual-level data were collected, household-level clustering (eg, siblings or cohabitants) was not tracked.

### COVID-19 Positivity Rates by Caza

**Figure 5.**
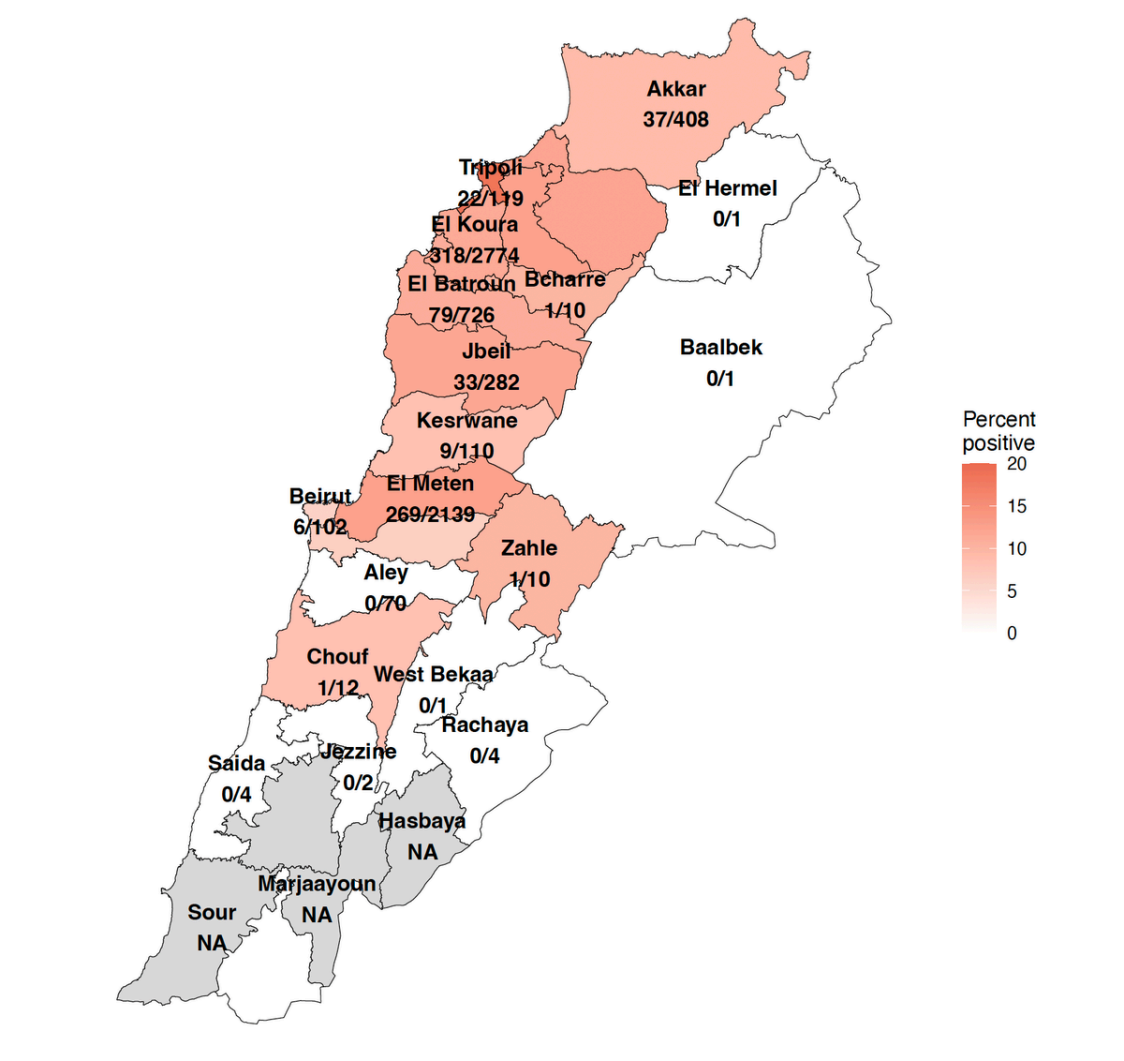
Geographical distribution of COVID-19 positivity rates across different cazas over any time during the survey period. Shaded areas indicate the positivity percentage, with darker shades denoting higher rates. Numerical overlays show the fraction of positive cases compared to total tests administered regionally.

We analyzed COVID-19 positivity rates based on participants’ reported caza of current residence, as shown in Figure 5. This variability likely reflects differences in viral spread and testing efficacy across regions. Notably, El Koura and El Meten reported the highest positivity rates among cazas with sufficient testing, at 11.4% (318/2774) and 12.5% (269/2139), respectively, suggesting they are focal points of infection. Tripoli, meanwhile, exhibited the highest positivity rate at 18.5% (22/119). Akkar and El Batroun also showed considerable positivity rates of 9.1% (37/408) and 10.9% (79/726), indicating moderate transmission levels.

### Trends in COVID-19 Positivity Rates: An Analysis From April 2020 to March 2021

**Figure 6.**
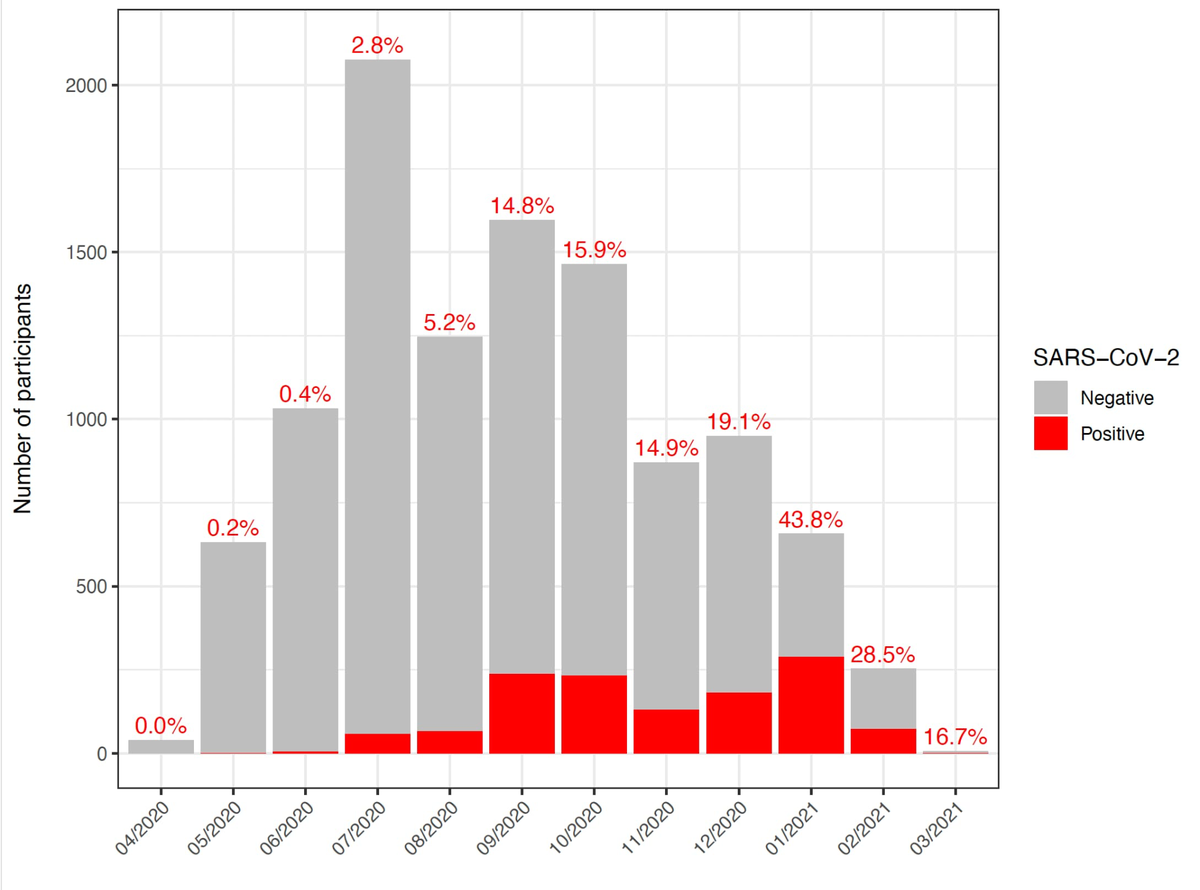
Monthly COVID-19 positivity trends. Stacked bars represent the total number of participants tested each month, divided into negative (gray) and positive (red) test results. The percentages atop each bar indicate the positivity rate for that month.

Between April 2020 and March 2021, COVID-19 positivity rates, derived from PCR-confirmed cases following submissions made through the HAYATI app, varied significantly, as shown in Figure 6. The rates started at negligible levels, gradually increased during the spring and early summer months, and showed a marked surge in the fall, peaking in October. After a slight decline in November, a sharp spike was recorded in January 2021, coinciding with the emergence of the Delta variant and representing the highest positivity rate observed during the study period. This was followed by a notable decline through March 2021. A similar temporal trend was observed in the official COVID-19 case data published by the Ministry of Public Health (MoPH).

### Symptom Distribution Among Individuals Screened for COVID-19

**Figure 7.**
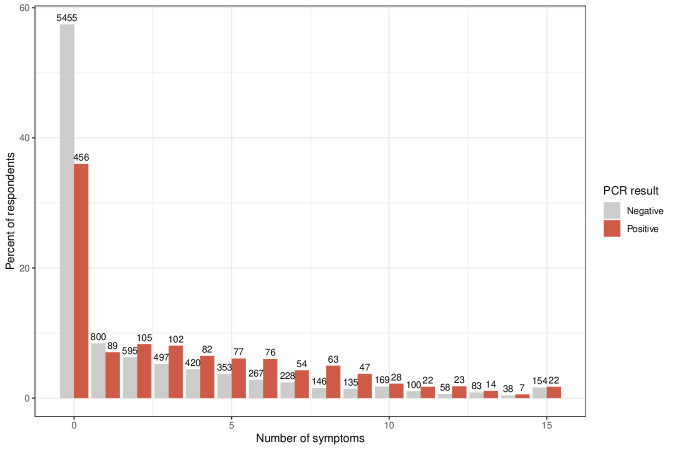
Symptom frequency in SARS-CoV-2 polymerase chain reaction (PCR) results. Bar plot of the number of symptoms reported by individuals with positive and negative SARS-CoV-2 PCR results.

**Figure 8.**
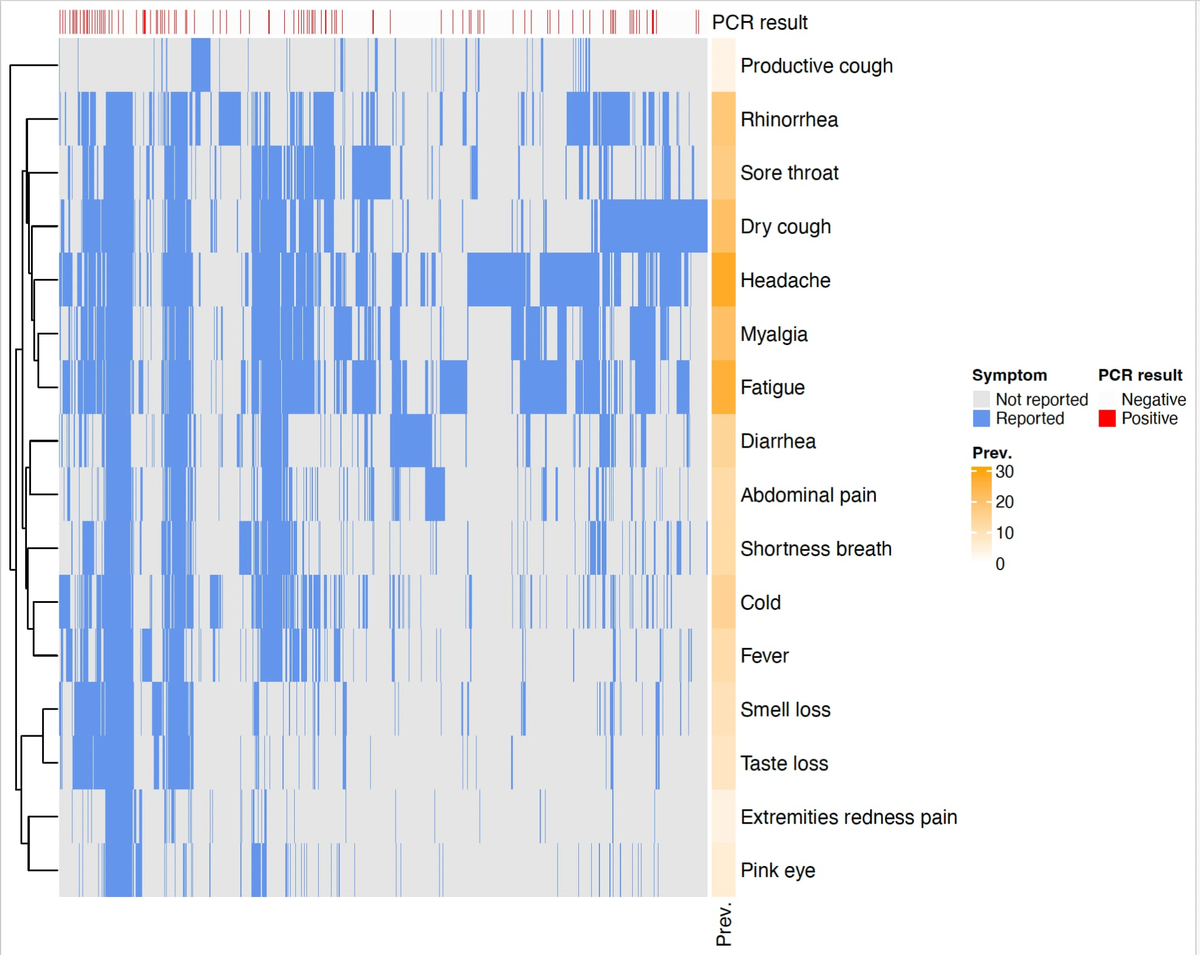
Overview of the co-occurrence of surveyed symptoms with SARS-CoV-2 PCR test results. Each row represents a different symptom, and the columns are individual participants, from any time during the survey period. Blue indicates that the participants reported the symptom, while grey indicates they did not report having the symptom. The overall percent prevalence (‘Prev.’) of each symptom is indicated in orange on the right side.

The analysis of symptoms was based on self-reported data collected through the HAYATI app and linked to PCR test results. Individuals presenting at the testing center exhibited a range of symptoms, leading to assessments for COVID-19. Notably, symptom presence was not a requirement for participation, allowing for the inclusion of asymptomatic individuals in the study. Among those who reported symptoms via the app, fever was recorded in 1273 individuals, with a corresponding positivity rate of 23%. Headaches and sore throats, reported in 3058 and 1844 cases, respectively, each had a positivity rate of 17%. Additionally, the loss of smell and taste was reported by 1065 and 914 individuals, respectively, correlated with a higher positivity rate of 25%. Figure 7. Our analysis demonstrates that loss of smell and taste was more strongly associated with positive COVID-19 test results than other common symptoms such as fever, myalgia, and cold. This correlation is illustrated in Figures7  8, which visualize the distribution and frequency of self-reported symptoms within the tested cohort. Figure 8 reveals distinct symptom clusters that are more commonly observed in PCR-positive cases.

Further exploration of the HAYATI app data examined the relationship between the number of reported symptoms and test positivity (Figure 7). A trend of increasing positivity rates was observed as the number of symptoms increased, particularly from 3 symptoms onward.

**Figure 9.**
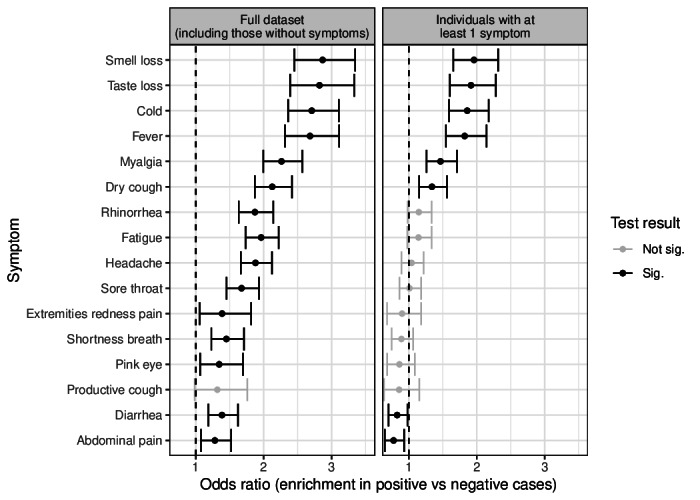
Intersection analysis of symptoms in positive and negative cases. Summary of reported symptoms enriched in participants with positive versus negative test results. Summary of reported symptoms enriched in participants with positive versus negative test results. The odds ratios based on a Fisher exact test are indicated for each individual symptom (bars indicate 95% CIs). Odds ratios >1 are enriched in positive cases. The left panel shows the odds ratio for the full dataset, including asymptomatic individuals, who are much more common among participants who tested negative. The right panel shows the same analysis restricted to participants with at least one symptom. Black points signify statistically significant differences (Benjamini-Hochberg corrected *P* values <.05) in symptom prevalence between positive and negative cases. The dotted line indicates an odds ratio of 1. Sig: significant.

**Figure 10.**
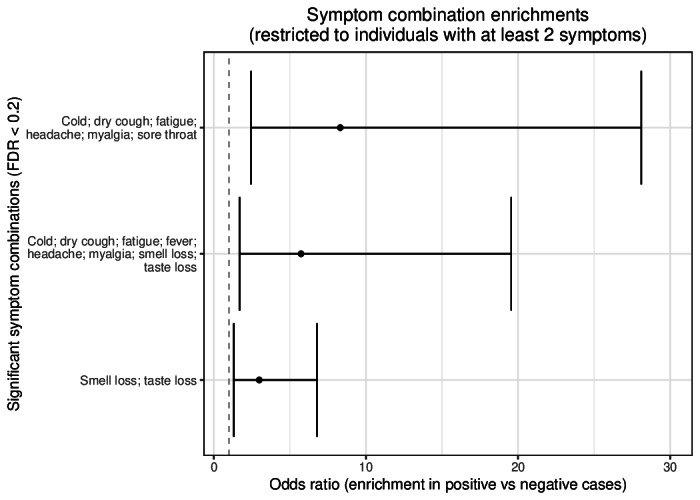
Enriched symptom combinations in COVID-19 positive cases. Significantly enriched symptom combinations in individuals with COVID-19 compared to individuals who tested negative (based on Fisher exact tests). Only individuals with at least 2 symptoms and symptom combinations that occurred at least 10 times were considered for this analysis. The plot illustrates the odds ratios for symptom combinations that are significantly more common in positive cases, with a Benjamini-Hochberg corrected *P* value <.2. The vertical bars represent the 95% confidence intervals for these ratios. The dotted line indicates an odds ratio of 1.

To evaluate the consistency of survey-based symptom reporting with known clinical patterns, we assessed symptom enrichment among positive cases (Figure 9). Significant symptom sets, including fever, myalgia, and notably, smell and taste loss, were markedly enriched in PCR-positive individuals. Analysis of specific symptom combinations further revealed elevated odds ratios for patterns involving respiratory and systemic symptoms such as cold, dry cough, and myalgia (Figure 10).

**Figure 11.**
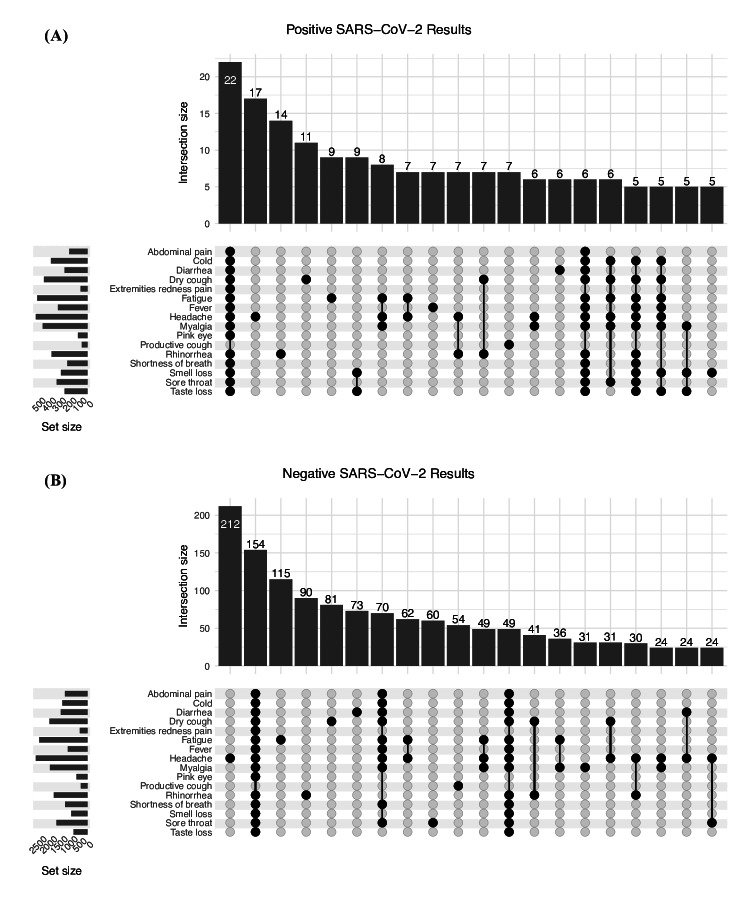
Intersection analysis of COVID-19 symptoms in positive and negative cases. The figure juxtaposes the symptom profiles of individuals with positive versus negative SARS-CoV-2 results using an UpSet plot format. Panel (A) focuses on the symptom intersections within the positive cases, while panel (B) does the same for the negative cases. The vertical bars represent the number of individuals reporting each intersection of symptoms, with the connected dots below delineating the specific symptoms within each intersection. This visualization elucidates the common symptom combinations in both cohorts (restricted to the top 20 most common symptoms in each case). The number of participants with each individual symptom is indicated by the horizontal bars on the left.

**Figure 12.**
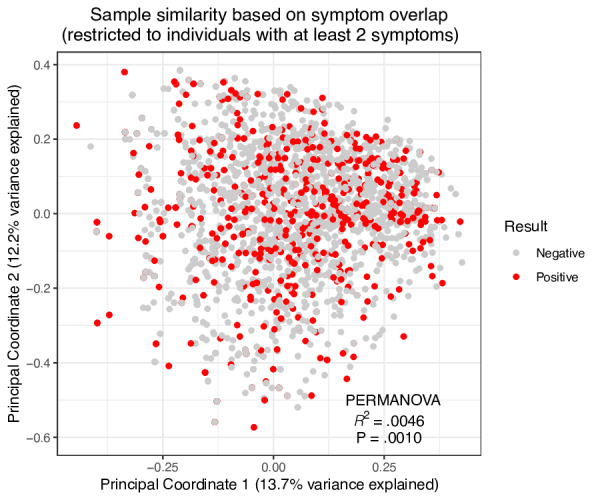
Symptom overlap and SARS-CoV-2 test results of principal coordinate analysis (PCoA). PCoA plot illustrating the similarity in symptom reporting between individuals with at least 2 symptoms, segmented by their SARS-CoV-2 polymerase chain reaction (PCR) test results. Each point represents a participant, with red indicating a positive test result and gray indicating a negative result. The proximity of points suggests the degree of similarity in symptoms reported. The effect size and *P* value of a PERMANOVA (permutational multivariate analysis of variance) statistical test of whether the PCR test result explains variation across samples is indicated.

Additionally, the UpSet plots (Figure 11) derived from HAYATI app data illustrate the frequency and co-occurrence of symptoms among PCR-positive and PCR-negative individuals, highlighting the predominance of smell and taste loss among those who tested positive. These patterns were further investigated through a principal coordinates analysis (Figure 12), which graphically represents individual cases based on symptom similarity. Although a statistically significant difference was detected (PERMANOVA: *R*²=0.0046, *P*=.001), the effect size was minimal, suggesting that while differences in symptom profiles between positive and negative cases exist, they explain only a small portion of the overall variation.

## Discussion

### Transforming Infectious Disease Management: The Hayati Project’s Role

The Hayati project was developed as a geospatial public health tool during a period of economic hardship, aiming to fill critical gaps in national surveillance by capturing data from individuals in underserved regions of North Lebanon. While national trends in COVID-19 cases were broadly reported by the Lebanese Ministry of Public Health, the HAYATI app provided several distinct advantages. First, the app enabled early, community-level surveillance by collecting self-reported symptoms and exposure data in near real-time, even before PCR confirmation. This type of participatory surveillance extended beyond the scope of MoPH systems, which primarily focused on confirmed case reporting. Hayati thus enabled proactive identification of potential hotspots before official case numbers increased. Second, and critically, the app was technically integrated with ArcGIS Pro, a professional-grade desktop GIS software. This allowed for automated spatial analysis, enabling researchers and public health officials to map the distribution of reported symptoms and confirmed cases, identify emerging clusters, and visualize epidemiologic trends in real time. This feature enhanced the utility of the data for rapid response and spatial decision-making, especially at the district (caza) level, where local municipalities could respond with targeted testing, outreach, or awareness efforts. Third, the initiative highlighted the role of a private academic institution in supporting national efforts through community-based, digital health tools. By offering free testing and building a regional digital infrastructure for data collection and spatial analysis, the University of Balamand demonstrated how academia can contribute actionable insights and public health resources during crises, particularly in resource-limited settings. Although some trends observed in the Hayati dataset aligned with national-level epidemiologic patterns, the value of the app lies in how the data were collected (community-driven), analyzed (spatially, via ArcGIS Pro), and acted upon (locally), providing operational support to public health authorities in a decentralized, real-time manner. A potential limitation of this study lies in the variability of self-reported data obtained through the app, particularly during the early phase of implementation when user compliance and reporting accuracy could not be consistently verified. This limitation is common in digital surveillance tools launched during public health emergencies and should be considered when interpreting risk profiles and exposure data.

### Alignment With Global COVID-19 Patterns

The patterns observed in the Hayati project are consistent with globally recognized characteristics of the COVID-19 pandemic, including variation in infection rates by age, symptom profile, and exposure-related behaviors. While these trends were broadly documented in national MoPH reports and international literature [13-17], the HAYATI app provided a community-driven, real-time dataset that mirrored these findings through self-reported inputs. For example, the strong association between loss of taste and smell and positive PCR results in our dataset reinforces the global understanding of these symptoms as key indicators of infection.

Although the Hayati project did not generate new epidemiological insights beyond those already known, it demonstrated that digital self-reporting platforms can effectively capture meaningful and reliable public health data, even in decentralized or resource-limited settings. The app’s ability to collect, visualize, and analyze spatially referenced data at the community level, particularly in underserved regions, offers a complementary tool to traditional surveillance systems.

Moreover, the implementation of the Hayati project during Lebanon’s economic and public health crisis serves as a case study for the feasibility and utility of digital health interventions in emergency contexts [18-20]. This model aligns with international efforts that highlight the value of early digital surveillance, public engagement, and spatial mapping to enhance pandemic preparedness and response [21-26].

### Demographic and Behavioral Associations With COVID-19 Positivity Rates

Our study reveals important insights into the associations between demographic factors, behaviors, and SARS-CoV-2 positivity rates. We observed a slight variance in positivity rates between genders, with females showing marginally higher rates than males. Contrary to findings from previous studies, significant disparities in positivity rates were observed across different blood types. Specifically, individuals with the O+ blood type were significantly less likely to test positive for COVID-19 compared to individuals with other blood types [27,28].

Our study reveals that behavioral factors, particularly adherence to social distancing and contact with confirmed cases, demonstrated a strong correlation with infection rates. Specifically, individuals who adhered to social distancing had a lower positivity rate of 9.9% compared to 13.0% for those who did not. Additionally, contact with COVID-19 positive patients was associated with a markedly higher positivity rate of 14.6%, in contrast to 10.0% for those without such contact.

Interestingly, several behaviors that we would expect to be associated with higher positivity rates were instead linked to lower positivity rates. For example, the use of public transportation was associated with a lower positivity rate of 8.2%, compared to 12.3% for those not using public transportation. Similarly, being in crowded areas was associated with a lower positivity rate of 8.9%, compared to 13.1% for those who avoided crowded areas. Recent travel was also negatively related to positivity rates, with recent travelers showing a significantly lower positivity rate of 2.2%, compared to 12.9% for those who had not traveled. Moreover, the rate of positivity was higher in people who did not have contact with travelers (12.2%) versus those who did (4.3%). Hospital visits were associated with a positivity rate of 9.0%, whereas those who had not recently visited a hospital had a higher rate of 12.0%. These counterintuitive findings may be explained by the context in which the data were collected, specifically during intermittent lockdowns and movement restrictions in Lebanon between April 2020 and February 2021. During these periods, individuals who traveled, used public transportation, or accessed public spaces were often essential workers or lower-risk individuals who adhered strictly to preventive measures such as mask-wearing, hand hygiene, and distancing. Conversely, people remaining at home may have been at increased risk of household transmission, which was a dominant mode of infection during lockdowns. Additionally, the possibility of uncontrolled confounding, such as differing levels of risk awareness or health status, must be considered. For instance, healthier individuals may have been more likely to engage in public activities while taking effective precautions. Finally, we acknowledge that all behavioral data were self-reported, which may introduce information and recall biases that could affect the accuracy of the observed associations.

Our data collection was not based on a random sample of the population, which introduces another layer of potential bias. Younger individuals, possibly more concerned about contracting COVID-19, may have been more likely to participate. This bias in participant demographics could affect the generalizability of our findings.

By recognizing these limitations, we can better understand the complexities of COVID-19’s transmission dynamics and further validate the critical role of public health guidelines.

The detailed analysis of how specific behaviors and demographic factors influence infection rates provides valuable insights for policymakers and public health officials in crafting targeted interventions to control the spread of the virus.

### Age-Specific Analysis and the Impact on Different Demographics

The age-specific analysis of COVID-19 positivity rates in our study reveals notable variation across age groups, with the highest number of positive cases observed among individuals aged 18-29 years. While this may reflect greater social activity or increased likelihood of seeking testing within this age group, it is essential to interpret these findings with caution. The study was based on a self-selected, nonrandom sample, which introduces potential selection bias. Younger individuals may have been more inclined to participate due to greater digital access, heightened health awareness, or perceived risk, which could influence the observed age distribution.

Moreover, participation in the study may have been shaped by socioeconomic and behavioral factors, such as access to mobile technology, willingness to engage with digital platforms, or the ability to reach testing centers. These potential confounders limit the generalizability of our findings to the broader population.

Given these limitations, the results should be viewed as indicative rather than representative, offering insights into symptom trends and positivity rates within a specific, engaged segment of the population. Future studies using randomized or more representative sampling strategies are needed to more accurately characterize COVID-19 transmission dynamics across different age groups.

### Conclusion

The study’s results highlight the impact of the Hayati project in deepening our understanding of COVID-19’s epidemiological characteristics within Lebanon. While the detailed analysis of demographic, behavioral, and clinical factors associated with SARS-CoV-2 positivity rates was conducted post-pandemic, it validates known global patterns and provides localized insights crucial for informed public health decision-making. This retrospective analysis underscores the potential benefits of real-time data analysis. Had these data been analyzed as they were collected, it could have significantly enhanced the responsiveness of health interventions during the pandemic. The successful application of GIS technology and self-reported data in tracking pandemic trends demonstrates the robustness and reliability of digital health tools. These tools hold promise for augmenting traditional epidemiological methods and offer valuable lessons for managing future health crises, particularly by leveraging real-time analysis to enable more timely and effective responses.

## Supplementary material

10.2196/80331 Multimedia Appendix 1 COVID-19 questionnaire variables and assigned weights in the HAYATI app.

10.2196/80331 Multimedia Appendix 2 HAYATI COVID-19 survey questionnaire by the University of Balamand GIS Center. This figure shows a part of the HAYATI COVID-19 survey questionnaire interface, developed by the GIS Center at the University of Balamand, as it appears while being filled out.

10.2196/80331 Multimedia Appendix 3 COVID-19 positivity rates by age group. The bar graph quantifies the percentage of positive cases within each age category (over all surveyed time periods). The absolute count of positive cases is indicated above each bar.

## Abbreviations

GIS: geographic information system
MoPH: Ministry of Public Health
PCR: polymerase chain reaction
PERMANOVA: permutational multivariate analysis of variance
