# Supplementary material for: Digital Survey–Based Tracing of COVID-19 Over the Early Pandemic: Comprehensive Geospatial and Symptomatic Analysis in Lebanon
Source: JMIR Public Health Surveill. 2025 Nov 20;11:e80331. doi: 10.2196/80331 (PMC12634038; doi:10.2196/80331)
Supplement: Multimedia Appendix 1 [file publichealth-v11-e80331-s001.docx]

| **Supplementary Table 1. COVID-19 Questionnaire Variables and Assigned Weights in the HAYATI App** Is this the first time you take the assessment or is it a retest? First time 🞏 Retest 🞏 |
| --- |
| Date & Time: |
| Household Location: |
| **Location of Household** |
| Governorate: |
| Caza: |
| Town: |
| **Contact Details** |
| Participant Number: |
| Date of Birth: |
| Age: |
| Gender: |
| Nationality: |
| Living alone? |
| Number of members living with you: |
| **Medical History** |
| Blood Type: |
| Chronic Disease: |
| Allergies: |
| Risk Factors: |

# Supp

| **Questionnaire Item** | **Weight / Rule** | **Notes** |
| --- | --- | --- |
| Are you respecting social distancing within the last 14 days? | — | Behavioral factor, not scored |
| Are you a healthcare worker, or laboratory worker handling specimens from a COVID-19 case? | — | Collected for surveillance, not scored |
| Do you wear the internationally recommended personal protective equipment (PPE)? | — | Behavioral factor, not scored |
| Have you used public transportation within the last 14 days? | 2 | Group 3 weighted factor |
| Have you been in crowded/closed areas within the last 14 days? | 2 | Group 3 weighted factor |
| Have you been in close contact with anyone who has returned from a travel trip within the last 14 days? | High risk (automatic) | Group 1 criterion |
| Have you had close contact with or cared for confirmed or suspected COVID-19 within the last 14 days? | High risk (automatic) | Group 1 criterion |
| Have you had face-to-face contact with COVID-19 case within 2 meters for more than 15 minutes? | High risk (automatic) | Group 1 criterion |
| Have you had physical contact with COVID-19 case? | High risk (automatic) | Group 1 criterion |
| Have you had unprotected direct contact with infectious secretions of a COVID-19 case? (e.g. being coughed on) | High risk (automatic) | Group 1 criterion |
| Were you in a closed environment (household, classroom, meeting room, etc.) with a COVID-19 case for more than 15 minutes? | High risk (automatic) | Group 1 criterion |
| Have you been to a hospital, as patient or visitor, within the last 14 days? | 2 | Group 3 weighted factor |
| Have you lost your sense of smell within the last 14 days? | High risk (automatic) | Group 1 criterion |
| Have you lost your sense of taste within the last 14 days? | High risk (automatic) | Group 1 criterion |
| Have you had runny nose within the last 14 days? | 1 | Group 3 weighted factor |
| Have you had dry cough within the last 14 days? | PCR referral if ≥ 2 present | Group 2 criterion |
| Have you had productive cough within the last 14 days? | 1 | Group 3 weighted factor |
| Have you had colds within the last 14 days? | 1 | Group 3 weighted factor |
| Have you had diarrhea within the last 14 days? | 1 | Group 3 weighted factor |
| Have you had sore throat within the last 14 days? | PCR referral if ≥ 2 present | Group 2 criterion |
| Have you experienced myalgia or body aches within the last 14 days? | 2 | Group 3 weighted factor |
| Have you had headache within the last 14 days? | 1 | Group 3 weighted factor |
| Have you had fever within the last 14 days? | PCR referral if ≥ 2 present | Group 2 criterion |
| Have you had difficulty breathing within the last 14 days? | PCR referral if ≥ 2 present | Group 2 criterion |
| Have you experienced fatigue within the last 14 days? | 2 | Group 3 weighted factor |
| Have you experienced abdominal pain within the last 14 days? | 2 | Group 3 weighted factor |
| Have you experienced sudden painful red spots on the body skin with swelling and redness of fingers or toes within the last 14 days? | — | Collected for monitoring, not scored |
| Have you had Pink/Red Eyes (Conjunctivitis) within the last 14 days? | — | Collected for monitoring, not scored |

This table summarizes the questionnaire used in the HAYATI application, covering participants’ health, exposures, and COVID-19 related symptoms. Group 1 criteria (critical exposures and hallmark symptoms) automatically classified respondents as high risk and required PCR testing. Group 2 criteria (key clinical symptoms) triggered PCR referral if two or more were present. Group 3 criteria (additional epidemiological and clinical factors) were weighted 1-3 points, with cumulative scores stratifying respondents into Minor (0-2), Moderate (2-6), Major (6-12), and High (12-20). Other items were collected for behavioral or epidemiological monitoring but were not included in the scoring algorithm.
