# Supplementary figures and images for: Digital Survey–Based Tracing of COVID-19 Over the Early Pandemic: Comprehensive Geospatial and Symptomatic Analysis in Lebanon
Source: JMIR Public Health Surveill. 2025 Nov 20;11:e80331. doi: 10.2196/80331 (PMC12634038; doi:10.2196/80331)

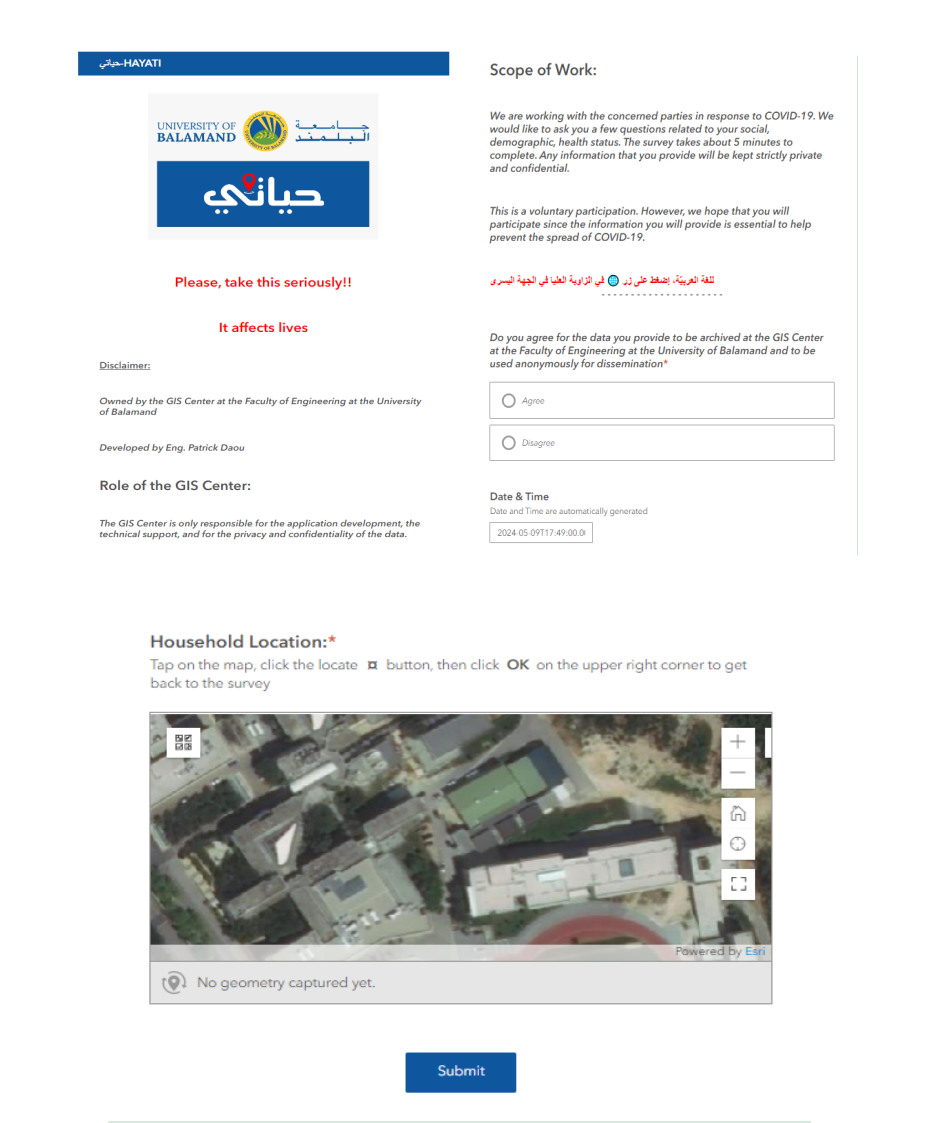

Supplement: Multimedia Appendix 2 [file publichealth-v11-e80331-s002.png]

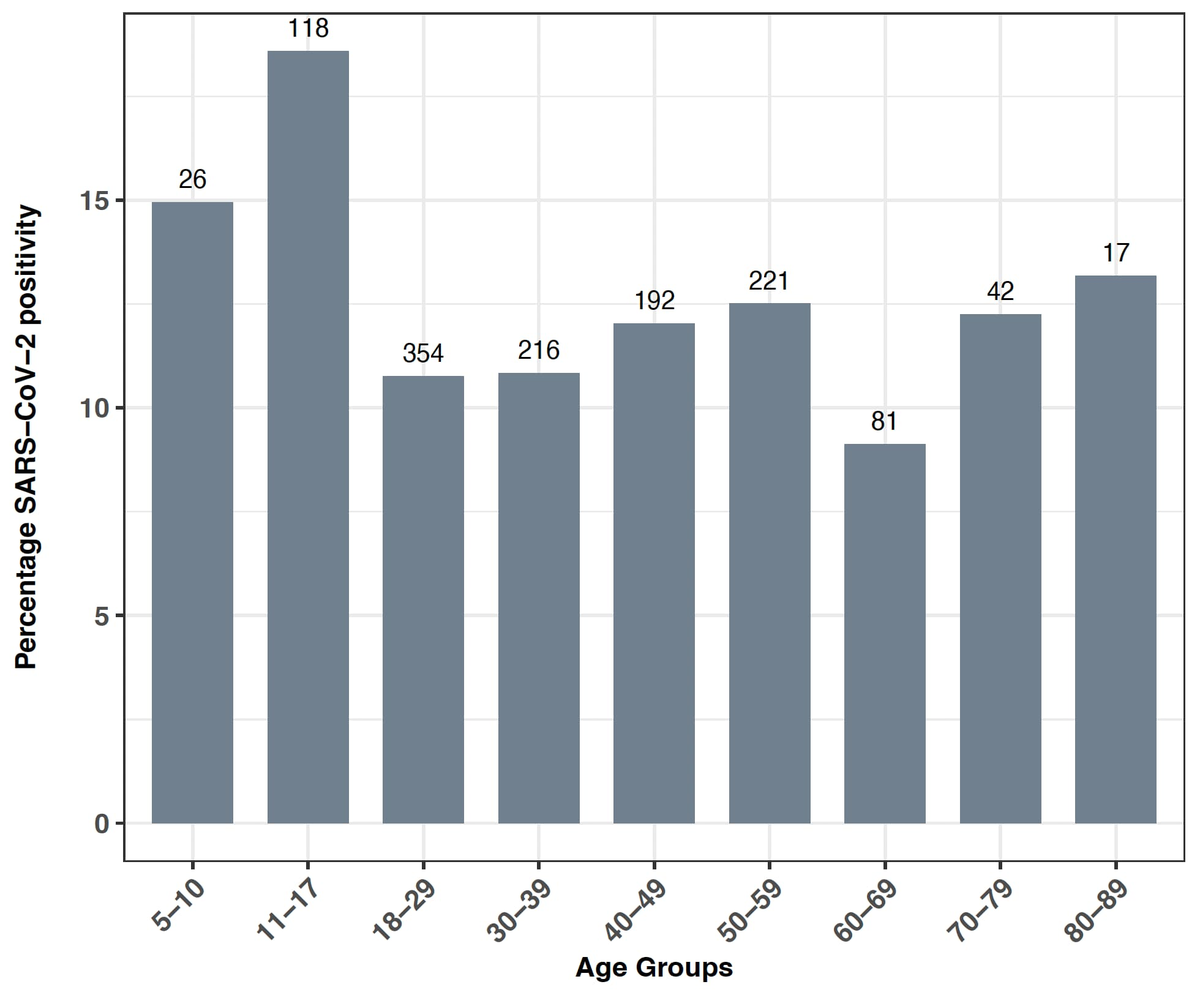

Supplement: Multimedia Appendix 3 [file publichealth-v11-e80331-s003.png]
